# Advances in technology and techniques for transcatheter aortic valve replacement with concomitant peripheral arterial disease

**DOI:** 10.3389/fmedt.2022.959249

**Published:** 2022-08-18

**Authors:** Chun-Ka Wong, Alston Conrad Ho-On Chiu, Kwong-Yue Eric Chan, Shu-Yue Sze, Frankie Chor-Cheung Tam, Ka-Chun Un, Simon Cheung-Chi Lam, Hung-Fat Tse

**Affiliations:** Cardiology Division, Department of Medicine, School of Clinical Medicine, Li Ka Shing Faculty of Medicine, The University of Hong Kong, Pokfulam, Hong Kong SAR, China

**Keywords:** TAVR - transcatheter aortic valve replacement, PAD - peripheral arterial disease, aortic stenosis, vascular closure device, percutaneous transluminal angioplasty

## Abstract

Aortic stenosis (AS) is a prevalent disease affecting 3.7% of the adult population aged 65 or above. In the past, surgical aortic valve replacement (SAVR) was the only definitive therapy available for the treatment of severe AS. Owing to the invasive nature of open-heart surgery, patients with advanced age and frailty could not benefit from SAVR. The advent of transcatheter aortic valve replacement (TAVR) in the past decade has offered an alternative treatment option for patients with severe AS, particularly those who are deemed to have high surgical risks. Nevertheless, a large proportion of patients also have concomitant peripheral arterial disease (PAD), which increases the risk of peri-procedural vascular complication, and precludes the possibility of transfemoral TAVR owing to inadequate luminal size for delivery system deployment. In this review, the prevalence and outcome of TAVR patients with PAD will be discussed. Furthermore, novel technologies and techniques that enable TAVR to be safely performed using transfemoral or alternative access in patients with severe PAD will be reviewed.

## Introduction

Aortic stenosis (AS) is a prevalent disease affecting 3.7% of the adult population aged 65 or above (1). Common etiologies of AS include degeneration, rheumatic heart disease, and congenital bicuspid aortic valve (2). Severe AS if left untreated is associated with poor prognosis with 1-year all-cause mortality up to 50.7% (3). In the past, surgical aortic valve replacement (SAVR) was the only definitive therapy available for the treatment of severe AS. Owing to the invasive nature of open-heart surgery, patients with advanced age and frailty could not benefit from SAVR. The advent of transcatheter aortic valve replacement (TAVR) in the past decade offered an alternative treatment option for patients with severe AS, particularly those who are deemed to have high surgical risks (3–10). Initial clinical studies comparing TAVR with SAVR primarily focused on patients who were deemed at high surgical risks. A 1-year survival analysis from the initial PARTNER trial demonstrated non-inferiority of TAVR over SAVR in patients with high surgical risks (4). Subsequent studies have been conducted to evaluate the outcomes in patients with intermediate-to-low surgical risks who underwent TAVR and SAVR. The results from the Evolut Low-Risk trial and PARTNER 3 trial once again demonstrated the non-inferiority of TAVR when compared to SAVR (9, 10). Furthermore, it was shown that among patients with intermediate-to-low risks, those who underwent TAVR had lower risks of bleeding, acute kidney injury, and atrial fibrillation than those who underwent SAVR (10). Nonetheless, SAVR still plays a key role in the management of patients with severe aortic diseases, especially among patients who are young, with age <55 years, and those requiring concurrent surgical valvular intervention or coronary artery bypass graft surgery (11). Certain anatomical features, such as low coronary ostia heights and shallow sinus of Valsava, would render TAVR a higher risk procedure (12, 13). Other advantages of SAVR include a lower pacemaker implantation rate and a lower incidence of postoperative aortic regurgitation (10).

Transcatheter aortic valve replacement has been increasingly performed worldwide and is commonly performed using transfemoral access (14). Nevertheless, a large proportion of patients also have concomitant peripheral arterial disease (PAD), which increases the risk of peri-procedural vascular complication, as well as precluding the possibility of transfemoral TAVR owing to inadequate luminal size for delivery system deployment. In this review, the prevalence and outcome of TAVR patients with PAD will be discussed. Furthermore, novel technologies and techniques that enable TAVR to be performed using transfemoral or alternative access in patients with severe PAD will be reviewed.

## TAVR patients with concomitant PAD

The peripheral arterial disease was present in a large proportion of patients who received TAVR. In the United States, 43.4% of patients from the STS/ACC TVT registry had PAD (15). Similarly, 50.0% of patients from the FRANCE TAVI registry and 25.1% of patients from the German Transcatheter Aortic Valve Interventions Registry had PAD (16, 17). Patients with concomitant PAD have a higher prevalence of cardiovascular risk factors, including hypertension (15), diabetes mellitus (15, 18), coronary artery disease (15), coronary revascularization (18), and prior stroke (15).

Patients with concomitant PAD have worse clinical outcomes after TAVR when compared to those without. European System for Cardiac Operative Risk Evaluation (EuroSCORE) II and Society of Thoracic Surgeons (STS) scores are two commonly used scoring systems for predicting the perioperative risk of TAVR patients. In the Korean TAVR registry, patients with PAD had a higher EuroSCORE II (10.4 vs. 4.16) and an STS score (8.83 vs. 6.23) when compared to patients without PAD (18). Transfemoral TAVR involves the usage of a large bore arterial sheath and delivery system through the iliofemoral system. As a result, patients with significant PAD had a higher prevalence of vascular complications. Real-world data from Korea revealed that the risk of major vascular complications was significantly higher among patients with PAD than those without (11.1 vs. 1.3%) (18). Beyond complications occurring at the vascular access site, patients with PAD also had a higher incidence of bleeding complications (23.1 vs. 19.7%) and even 1-year mortality (16.8 vs. 14.4%) (15).

## Anticoagulation strategy

There have been studies examining the use of alternative peri-operative anticoagulation strategies to minimize vascular complications and bleeding risk among TAVR patients with PAD. In the BRAVO-3 trial, it was found that among patients with PAD, anticoagulation with bivalirudin does not reduce the risk of bleeding or vascular complication when compared to unfractionated heparin, which is the standard anticoagulation used in most centers (19). Among patients who have an alternative indication for vitamin K antagonist, it was found that uninterrupted warfarin therapy among patients with PAD was associated with a drastically high risk of vascular complication when compared to those who had no PAD, with a relative risk of 10.95 (20).

## Vascular closure devices

As transfemoral TAVR involves the use of large bore arterial sheaves and delivery systems, it is necessary to apply vascular closure devices to the arteriotomy site for hemostatic purposes. Percutaneous vascular closure devices used in TAVR procedures can be broadly categorized as suture-based and plug-based. Suture-based devices such as Perclose ProGlide System (Abbott, USA) percutaneously deliver sutures to the arteriotomy site for vascular closure. Plug-based devices such as ANGIO-SEAL (Terumo, Japan) and MANTA (Teleflex, USA) achieve hemostasis by delivering a collagen plug to the arteriotomy site.

The choice of vascular closure strategy is influenced by the severity and the pattern of PAD in the utilized artery. The chances of failing to deploy a suture-based vascular closure device are higher in vessels with severe anterior wall calcification, as the percutaneously delivered suture may fail to oppose the arteriotomy vessel wall. Nevertheless, despite optimal patient selection, the risk of major vascular complications among patients treated with suture-based vascular closure devices is still higher among patients with PAD when compared to those without, with an odds ratio of 3.28 (21). On the other hand, it may also be challenging to successfully implant plug-based devices in the presence of severe iliofemoral PAD, for instance, due to failure of footplate deployment. In a case series comprising 100 patients treated with MANTA devices, which is a plug-based device specifically designed for the closure of large bore femoral arteriotomy sites, concomitant PAD increases the proportion of patients with vascular closure device-related complications from 13.5 to 45.5% (22).

## Transfemoral TAVR

Transfemoral access is the most commonly employed strategy for TAVR. One key prerequisite of performing transfemoral TAVR is to have a sufficient iliofemoral luminal size to allow the passage of large bore TAVR sheaves and delivery systems. Patients with PAD have stenotic iliofemoral arteries and calcific vessel walls that are prone to develop vascular complications. Vascular access can be comprehensively assessed by a pre-operative computed tomography. The main parameters that will be assessed include minimal luminal diameter along the access route, vessel tortuosity, and vessel calcification (23). The use of novel diagnostic and therapeutic tools allow transfemoral TAVR to be safely performed in patients with PAD.

Before the operation, it is necessary to determine whether the iliofemoral system has sufficient caliber to accommodate the delivery system. Key parameters that will be reported in computed tomography scans include minimal and mean diameter at bilateral common femoral, external iliac, and common iliac arteries. In addition to relying on the minimum vessel diameter recommended by the manufacturer, researchers have also developed risk scores to predict the risk of vascular complications from transfemoral TAVR. In the early days of TAVR, a sheath-to-femoral ratio (SFAR) threshold of 1.05 was found to be highly predictive of vascular complications, with sensitivity, specificity, positive predictive value, negative predictive value, and area under the receiver–operator characteristic curve of 66.7, 65.6, 40.7, 84.7, and 0.727%, respectively. Furthermore, it was determined that the optimal SFAR threshold for calcific and non-calcific vessels were 1.0 and 1.1, respectively (24). Nevertheless, as modern delivery systems have a smaller caliber in comparison to the larger systems used in the aforementioned study (Table 1), a modified version of SFAR, namely md-SFAR, was established to improve predictive capabilities. The md-SFAR threshold is device-specific and prosthetic heart valve size-specific. For instance, md-SFAR cut-offs for Evolut R (Medtronic, USA) 14F sheath, Evolut R 16F sheath, Sapien 3 (Edwards Lifesciences, USA) 23 mm 14 F sheath, and Sapien3 29 mm 16F sheath are 1.2, 1.22, 1.09, and 1.12, respectively (25).

**Table 1:** Caliber of transcatheter aortic valve replacement systems.

**Transfemoral TAVR systems**	**Labeled delivery system diameter (F)**
Sapien 3 (Edwards Lifesciences, USA)	14F, 16F
Evolut PRO+ (Medtronic, USA)	14F, 18F
Evolut R (Medtronic, USA)	14F, 16F
Navitor (Abbott, USA)	14F, 15F
Acurate neo2 (Boston Scientific, USA)	14F
ALLEGRA (New Valve Technology, Switzerland)	15F

*TAVR, transcatheter aortic valve replacement*.

Qualitative grading of vascular tortuosity can be assessed in the pre-operative computed tomography using a three-point scale ranging from mild, moderate, to severe (23). More recently, novel computed tomography software packages enable more refined vascular complication risk prediction. Automated tracking of arteries and measurement of angles between contiguous segments allow the quantification of iliofemoral tortuosity for vascular complication risk prediction (26). It has also been shown that by using dedicated software for quantifying iliofemoral artery lumen volume and artery wall volume, it is possible to enhance predictive capabilities for vascular complications (27).

## Angioplasty and intravascular lithotripsy-assisted transfemoral TAVR

In patients with severe PAD and insufficient iliofemoral diameter for transfemoral TAVR, percutaneous transluminal angioplasty and stenting may be performed to increase the luminal profile to allow the passage of the TAVR delivery system (28). According to data extracted from Nationwide Readmissions Database from the United States, 4.42% of patients who underwent TAVR from 2016 to 2017 had peripheral vascular intervention performed in the same admission. Peripheral vascular intervention-assisted TAVR was found to have a superior outcome when compared to TAVR performed using non-femoral alternative access, with a lower risk of mortality (3.0 vs. 4.6%), acute kidney injury (14.5 vs. 22.7%), 30-day readmission (15.5 vs. 18.1%), and shorter length of stay (4 vs. 5 days) (29). Some patients may have calcific PAD lesions that cannot be adequately dilated with conventional balloon angioplasty. In this subgroup of patients, intravascular lithotripsy systems, such as Shockwave Intravascular Lithotripsy (Shockwave Medical, USA), can be utilized. When the intravascular lithotripsy system is activated, electrical discharge vaporizes fluid within the balloon catheter to create rapidly expanding and collapsing bubbles, which in turn generate sonic waves that crack intimal and medial calcium in the PAD vessel walls (30). Intravascular lithotripsy is more commonly delivered to common and external iliac arteries using a catheter with a 6.0–7.0 mm diameter (30, 31). Patients with focal calcific stenosis <20 mm in length requires a minimal luminal diameter of ≥4.0 and ≥3.0 mm for calcified lesions with a circumference of 360° and 270%, respectively. On the other hand, patients with diffuse calcific stenosis >20 mm in length require a minimal luminal diameter of ≥4.5 and ≥3.5 mm for calcified lesions with a circumference of 360° and 270%, respectively (32). In a European TAVR registry, intravascular lithotripsy utility increased from 2.4% in 2018 to 6.5% in 2020. Among the 108 patients treated with intravascular lithotripsy, the success rate of transfemoral delivery of the TAVR system was 100%, and the rate of vascular complication was low with 0.93% and 2.78% in patients having vascular perforation and dissection, respectively (31).

## Alternative access TAVR

**Figure 1:**
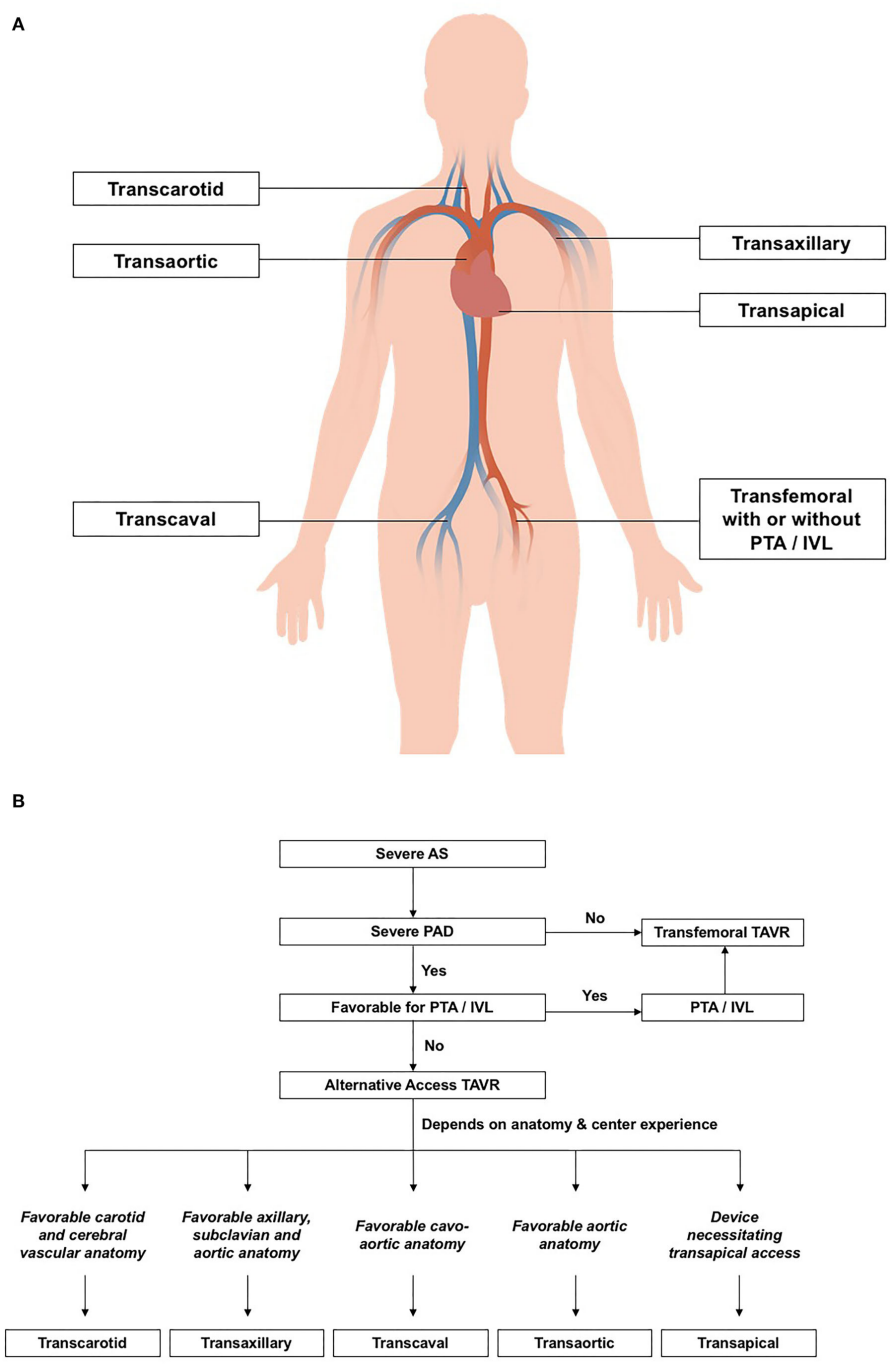
**(A)** Transcatheter aortic valve replacement (TAVR) strategies in patients with concomitant peripheral arterial disease. **(B)** Anatomical factors for deciding vascular access for TAVR. AS, aortic stenosis; IVL; intravascular lithotripsy; PAD, peripheral arterial disease; PTA, peripheral transluminal angioplasty; SFAR, Sheath-to-femoral ratio; TAVR, transcatheter aortic valve replacement.

Despite percutaneous transluminal angioplasty and intravascular lithotripsy, a small proportion of patients may still have an inadequate iliofemoral luminal profile for transfemoral delivery of the TAVR system (14, 33). In this subgroup of patients, TAVR may need to be performed using non-femoral alternative access (Figure 1). In the FRANCE TAVI registry involving 21,611 patients, 7.5% received TAVI *via* non-femoral approaches (16). Among the array of alternative vascular accesses for TAVR, transaxillary and transcarotid approaches were most commonly used. Other non-femoral options include transcaval, transapical, and transaortic approaches (14, 33, 34). Comparison between alternative vascular access in terms of the odds ratio of mortality, stroke, vascular complication, and acute renal failure is summarized in Table 2.

**Table 2:** Comparison between alternative vascular access.

**Alternative TAVR access**		**Odds ratio** ^**#**^				**Refrences**
		**Mortality**	**Stroke**	**VC**	**AKI**	
Transaxillary (*n* = 1,180)	Transapical/transaortic (*n* = 1,180)	0.6^*^	2.1^*^	0.28	1.46^*^	(35)
Transaxillary (*n* = 19)	Transapical (*n* = 16)	0.39	2.68	–	–	(36)
Trans-subclavian (*n* = 188)	Transapical (*n* = 761)	0.23^*^	1.06	5.49^*^	0.51	(37)
	Transaortic (*n* = 185)	0.31^*^	6.07	0.65^*^	0.33^*^	
Trans-subclavian (*n* = 60)	Transapical (*n* = 142)	0.18	2.41	1.01	0.72	(38)
Trans-subclavian (*n* = 11)	Transapical/transaortic (*n* = 22)	2.1	–	–	–	(39)
	Transapical (*n* = 11)	1	–	–	–	
	Transaortic (*n* = 11)	3.29	–	–	–	
Trans-subclavian (*n* = 17)	Transcarotid (*n* = 43)	–	–	1.28	–	(40)
	Transaortic (*n* = 67)	–	–	0.64	–	
	Transapical (*n* = 45)	–	–	0.5	–	
	Transaortic/transapical (*n* = 112)	–	–	0.57	–	
Transcarotid (*n* = 43)	Transaortic (*n* = 67)	0.4275	0.51	0.5	0.51	
	Transapical (n = 45)	1.61	0.33	0.39	0.24	
	Transaortic/transapical (*n* = 112)	0.625	0.42	0.45	0.36	
Transcarotid/transubclavian (*n* = 87)	Transaortic/transapical (*n* = 104)	0.58	0.29	2.42	–	(41)
Transcarotid (*n* = 49)	Transapical (*n* = 53)	0.53	–	1.09	–	(42)
Transcarotid (*n* = 84)	Transaortic (*n* = 33)	0.38	0.78	–	–	(43)
	Transapical (*n* = 48)	1.15	1.15	–	–	
	Transapical/transaortic (*n* = 81)	0.63	0.96	–	–	
Transcarotid (*n* = 94)	Transapical/transaortic (*n* = 163)	0.45	0.6	0.5	–	(44)
Transcarotid (*n* = 788)	Transaxillary (*n* = 1576)	0.79	0.54^*^	0.68	2.21	(45)
Transcaval (*n* = 238)	Transaxillary (*n* = 106)	0.88	0.2^*^	2.72	0.42	(46)

# *Odds ratio were calculated by chi-square test using data from the corresponding publication*.

* *p-value < 0.05*.

*AKI, acute kidney injury; TAVR, transcatheter aortic valve replacement; VC, vascular complications*.

## Transaxillary and trans-subclavian TAVR

Transaxillary and trans-subclavian TAVR are the most commonly utilized non-femoral TAVR access in the United States (14). It is advantageous to utilize the axillary artery for TAVR in patients with PAD because, unlike the iliofemoral system, there is usually a relative lack of atherosclerosis in the axillary arteries. In the past, surgical cut down is required to perform transaxillary and trans-subclavian TAVR. Recently, it is predominantly performed using percutaneous approaches (47). At the beginning of the procedure, an arterial puncture is performed under sonographic and fluoroscopic guidance. The left axillary artery is usually utilized as it allows more favorable alignment with the ascending aorta and aortic valve annulus. It is followed by aortic valve crossing, stiff wire exchange, serial dilatation, and TAVR delivery system deployment. During serial dilatation of arteriotomy site and vascular closure deployment, balloon tamponade at the proximal axillary artery segment is performed to reduce blood loss.

Perioperative stroke risk is relatively higher among patients receiving transaxillary and subclavian TAVR when compared to other non-femoral approaches. Data from the STS/ACC TVT registry comprising 1,180 transaxillary and trans-subclavian TAVR procedures revealed a stroke risk of 6.3%, which was significantly higher than transthoracic approaches with an odds ratio of 2.1 (35). Similarly, transaxillary and trans-subclavian TAVR have been shown to be associated with higher stroke risks when compared to transcarotid and transcaval approaches (45, 46) (Table 2).

## Transcarotid TAVR

Transcarotid TAVR is the second most commonly utilized alternative vascular access in the United States (14). At the beginning of the procedure, a surgical cut-down is performed to gain exposure to the common carotid artery, which is followed by establishing proximal and distal control of the common carotid artery using techniques similar to carotid endarterectomy procedures. After insertion of a small arterial sheath, the aortic valve is crossed with a guidewire and diagnostic catheter. Stiff wire exchange will then be performed, followed by serial dilatation and TAVR delivery system deployment. At the end of the procedure, the arteriotomy site is repaired by sutures (34, 48).

As arterial supply through the ipsilateral carotid artery will be temporarily interrupted during the transcarotid TAVR, it is crucial to ensure the absence of severe stenosis in the contralateral carotid artery before operation using duplex ultrasound. Furthermore, some centers perform routine MRI to ensure the integrity of the Circle of Willis. Among 788 patients from the STS/ACC TVT registry who received transcarotid TAVR between 2015 and 2019, it was found that the tranascarotid approach was associated with a lower stroke risk than transaxillary approach, with an odds ratio of 0.54 (*p* = 0.003^*^) (45) (Table 2).

## Transcaval TAVR

Transcaval TAVR *via* caval-aortic access is reserved for patients who have no alternative access to TAVR procedures. Novel techniques and tools have been developed to enable this alternative TAVR approach. Pre-operative computed tomography is performed to identify an optimal caval-aortic crossing site with the closest distance between the two vessels and with the least aortic wall calcification. Fluoroscopic landmarks are accessed using vertebral levels as reference. During the procedure, a simultaneous aortogram and venogram are performed to confirm the target puncture site. Gooseneck snare is positioned in the aorta at the level of the anticipated crossing site. A crossing system consisting of stiff wire inside a wire convertor and a support catheter is introduced to the crossing site from the inferior vena cava. The guidewire is connected to a unipolar electrosurgery pencil using forceps to allow electrosurgical puncture of the two vessels. Thereafter, a guidewire and a catheter cross to the aorta and are snared. Stiff wire exchange is then performed, followed by serial dilatation and TAVR delivery system deployment (49). After the procedure, the cavo-aortic tract is closed using occluders originally designed for intracardiac defects. More recently, a dedicated transcaval closure device (TCD; Transmural Systems, USA) comprising a braided nitinol double-disc design with an interconnecting spring between the two intravascular discs and a retention paddle has been devised (50). In pilot studies involving human subjects, the dedicated TCD was shown to achieve complete closure of the cavo-aortic tract in 75% of patients at the end of the TAVR procedure and 100% on day 30 (50) (Table 2).

In a recently published propensity-weighted analysis involving 238 patients who received transcaval TAVR from eight experienced centers in the United States, it was shown that transcaval TAVR was superior to transaxillary access with respect to stroke risk with an odds ratio of 0.2 (*p* = 0.014). Furthermore, the trend toward lower mortality and acute renal failure risk was observed, and also statistical significance was not reached (46).

## Transapical and transaortic TAVR

**Figure 2:**
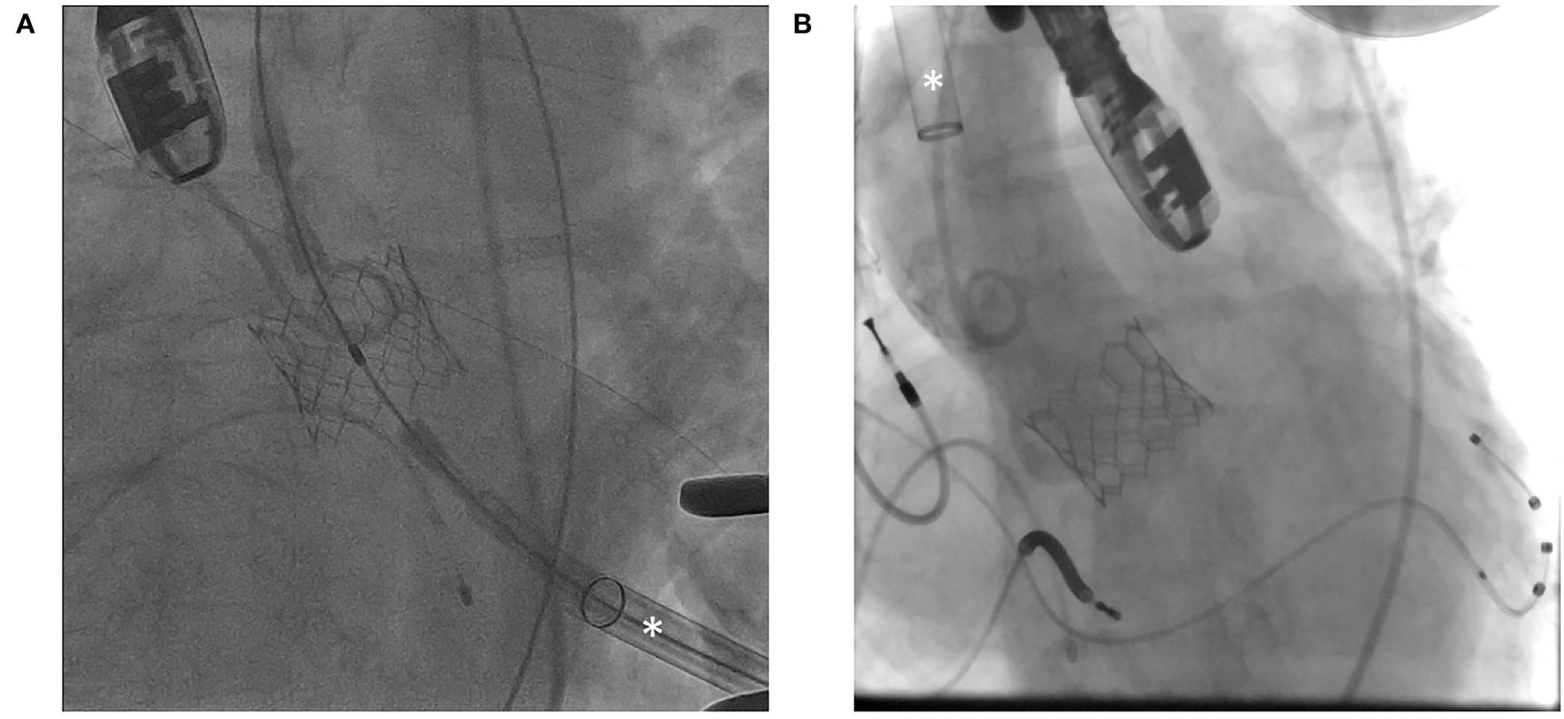
Fluoroscopic images demonstrating **(A)** transapical approach and **(B)** transaortic approach. Asterisk (*) indicates transcatheter aortic valve replacement (TAVR) delivery sheath.

Transapical and transthoracic accesses are two transthoracic approaches for performing TAVR in patients with severe PAD. Access to the cardiac apex is obtained using the left thoracotomy. Direct myocardial puncture adjacent to the true cardiac apex is performed after applying pledgeted sutures. It is followed by the crossing of the aortic valve, stiff wire exchange, and TAVR delivery system deployment (Figure 2A). At the end of the procedure, the myotomy wound is closed by the tightening of the pre-deployed sutures. In the past, closure devices for transapical TAVR have been devised (51), although they have not been widely adopted despite years of development. Transaortic TAVR is performed using right lateral sternotomy or J-sternotomy. Direct aortic puncture is performed after applying pledgeted sutures. Subsequently crossing of the aortic valve, stiff wire exchange, delivery system deployment, and aortic valve prosthesis implantation is performed (Figure 2B).

One key advantage of the transapical approach is the availability of TAVR systems designed specifically for pure aortic regurgitation, such as J Valve (JC Medical, USA). Furthermore, similar to other non-femoral approaches, transthoracic TAVR is associated with lower stroke risk than the transaxillary approach (35). Among 1,180 patients from the STS/ACC TVT registry who underwent propensity score matching, patients who received transapical or transaortic TAVR had lower stroke risk (3.1 vs. 6.3%), but higher 30-day mortality (8.4 vs. 5.3%) and longer hospital stay when compared to those who received transaxillary TAVR (35). In the UK, TAVI registry involving 946 patients, transapical and transaortic approaches were associated with mortality risk when compared to trans-subclavian TAVR (37) (Table 2).

## Suture-less and rapid deployment valves

Suture-less and rapid-deployment valves (SURDs) have emerged as an alternative minimally invasive option for patients with severe AS, especially for those with concurrent PAD, in whom TAVR could not be offered with transfemoral or alternative vascular access. In comparison to conventional SAVR, SURDs involve only three or fewer sutures for the anchorage of bioprosthesis within the aortic annulus (52). Current examples of SURDs include 3f Enable (Medtronic, USA), Perceval (LivaNova, United Kingdom), and Intuity (Edwards Lifesciences, USA). Each of these SURDs involve different numbers of sutures and relies on individualized mechanisms for operation. In contrast to TAVR, which involves a transcatheter approach, SURDs still require surgical incisions and excision of the diseased native valves. Nevertheless, it can be conducted in a minimally invasive manner *via* ministernotomy and minthoracotomy and with shorter cardiopulmonary bypass and cross-clamp durations. Different trials have been conducted to compare SURDs and conventional SAVR (53). In the CADENCE-MIS trial, it was demonstrated that those who received the Intuity SURD had significantly reduced aortic cross-clamp duration, shorter myocardial ischemic time, and reduced mean transvalvular gradient (54). Another clinical trial, namely PERSIST-AVR, demonstrated non-inferiority of Perceval SURD when compared to conventional SAVR in terms of major adverse cardiac and cerebrovascular events at 1 year. The surgical duration was reduced in the Perceval group when compared to conventional SAVR. Nonetheless, an increased rate of pacemaker implantation was observed in the Perceval group (55).

## Discussion

A high proportion of patients undergoing TAVR have concomitant PAD. It has been known that the presence of PAD is a predictor of worse peri-operative and long-term outcomes. Stenotic iliofemoral arteries may also pose technical challenges for transfemoral TAVR. The proportion of patients who can undergo transfemoral TAVR has been expanded by percutaneous luminal angioplasty and intravascular lithotripsy in recent years. In contemporary TAVR registries, only <5% of patients required the use of alternative vascular access (14). To date, it is controversial which is the most superior non-femoral access for TAVR. All currently available data are derived from TAVR registries, which are potentially limited by selection biases and other forms of confounding factors. As there has been no data from randomized trials that illustrate relative safety and efficacy among non-femoral approaches, the decision on which alternative access to utilize has to rely on the individualized decision of each center. Important considerations include relative expertise of each non-femoral approach by the heart team in concern and the patient factor including anatomical characteristics and frailty.

Currently, the key knowledge gap for managing TAVR patients with PAD is the relative safety and efficacy of the various non-femoral TAVR approaches. The important clinical question is ideally addressed by multi-center randomized controlled trials involving centers that have the expertise to perform TAVR using multiple alternative access. In terms of future development, devices and methods to reduce transaxillary TAVR stroke risk and dedicated cavo-aortic tract closure device for transcaval TAVR have to be further explored.

## Conclusion

There is a high prevalence of PAD among patients receiving TAVR. In addition to having worse peri-operative clinical outcomes, severe iliofemoral PAD also poses technical challenges for transfemoral TAVR. Novel technologies and techniques enable TAVR to be safely performed using transfemoral or alternative access in patients with severe PAD.

## Author contributions

C-KW, K-YC, SL, and H-FT contributed to the conception and design of the review article. C-KW, AC, K-YC, S-YS, FT, K-CU, SL, and H-FT performed data acquisition. C-KW, AC, and K-YC wrote the first draft of the manuscript. S-YS, FT, K-CU, SL, and H-FT revised the protocol critically for important intellectual content. All authors have read and approved the final version of the manuscript to be published.

## Conflict of interest

The authors declare that the research was conducted in the absence of any commercial or financial relationships that could be construed as a potential conflict of interest.

## Publisher's note

All claims expressed in this article are solely those of the authors and do not necessarily represent those of their affiliated organizations, or those of the publisher, the editors and the reviewers. Any product that may be evaluated in this article, or claim that may be made by its manufacturer, is not guaranteed or endorsed by the publisher.
